# Exercise for Hypertension: A Prescription Update Integrating Existing Recommendations with Emerging Research

**DOI:** 10.1007/s11906-015-0600-y

**Published:** 2015-09-30

**Authors:** Linda S. Pescatello, Hayley V. MacDonald, Lauren Lamberti, Blair T. Johnson

**Affiliations:** Department of Kinesiology, College of Agriculture, Health, and Natural Resources, University of Connecticut, 2095 Hillside Rd, U-1110, Storrs, CT 06269-1110 USA; Department of Psychological Sciences, College of Liberal Arts and Sciences, University of Connecticut, 406 Babbidge Rd, U-1020, Storrs, CT 06269-1020 USA

**Keywords:** Aerobic exercise, Blood pressure, Concurrent exercise, Postexercise hypotension, Prehypertension, Resistance exercise

## Abstract

Hypertension is the most common, costly, and preventable cardiovascular disease risk factor. Numerous professional organizations and committees recommend exercise as initial lifestyle therapy to prevent, treat, and control hypertension. Yet, these recommendations differ in the components of the Frequency, Intensity, Time, and Type (FITT) principle of exercise prescription (Ex R_x_); the evidence upon which they are based is only of fair methodological quality; and the individual studies upon which they are based generally do not include people with hypertension, which are some of the limitations in this literature. The purposes of this review are to (1) overview the professional exercise recommendations for hypertension in terms of the FITT principle of Ex R_x_; (2) discuss new and emerging research related to Ex R_x_ for hypertension; and (3) present an updated FITT Ex R_x_ for adults with hypertension that integrates the existing recommendations with this new and emerging research.

## Introduction

Hypertension is the most common, costly, and preventable cardiovascular disease (CVD) risk factor [1, 2]. Approximately 80 million Americans (33 %) have hypertension (systolic blood pressure [SBP] ≥140 mmHg and/or diastolic blood pressure [DBP] ≥90 mmHg), and another 87 million (36 %) have prehypertension (SBP ≥120–<140 mmHg and/or DBP ≥80–<90 mmHg); amounting to nearly 70 % of Americans with high blood pressure (BP) [1]. Projections indicate that by 2030 over 40 % of adults in the USA will acquire hypertension. From 2010 to 2030, the total direct costs attributed to hypertension are projected to triple from US$130.7 to US$389.9 billion, while the indirect costs due to lost productivity will almost double from US$25.4 to US$42.8 billion [2]. Lifestyle factors, such as participation in regular exercise, are recognized as key modifiable determinants of hypertension. Therefore, there is a need for more intensive efforts to promote these strategies to reduce the significant public health burden of hypertension [3].

Numerous randomized controlled trials (RCTs) have been conducted investigating the antihypertensive effects of exercise. In an attempt to better quantify the antihypertensive effects of exercise, many meta-analyses of these RCTs have been published [4••, 5••]. These meta-analyses concluded that aerobic exercise training lowers blood pressure (BP) 5–7 mmHg [6–8], while dynamic resistance training lowers BP 2–3 mmHg [6, 8–11] among adults with hypertension. The magnitude of these BP reductions rivals the magnitude of those obtained with first-line antihypertensive medications [12] and lower CVD risk by 20–30 % [13]. Exercising as little as 1 day per week is as effective (or even more so) than pharmacotherapy for reducing all-cause mortality among those with hypertension [14]. Furthermore, a recent network meta-analysis [15] of major exercise and drug trials showed no statistically detectable difference between exercise and drug interventions in mortality outcomes for coronary heart disease and prediabetes, and physical activity interventions were actually more effective for the secondary prevention of stroke mortality. For these reasons, the Joint National Commission (JNC) 7 [16], the JNC 8 [17] and American Heart Association (AHA)/American College of Cardiology 2013 Lifestyle Work Group [18], another recent AHA Scientific Statement [19], the American College of Sports Medicine (ACSM) [6], the European Society of Hypertension and European Society of Cardiology (ESH/ESC) [20], and the Canadian Hypertension Education Program (CHEP) [21] all recommend exercise for the prevention, treatment, and control of hypertension (Table 1).

**Table 1:** The existing professional exercise recommendations among adults with hypertension [5••]

Professional Committee/Organization						
The *FITT* of the exercise prescription	Joint National Committee, 8th Report [17] and the AHA/ACC Lifestyle Work Group [18]	Joint National Committee, 7th Report [16]	American Heart Association [19]	American College of Sports Medicine [6]	European Society of Hypertension/ European Society of Cardiology [20]	Canadian Hypertension Education Program [21]
Frequency (how often?)	3–4 sessions⋅week^−1^ ≥ 12 weeks	Most days of the week	Most days of the week	Most, preferably all, days of the week	5–7 days⋅week^−1^	4–7 days⋅week^−1^ in addition to habitual, daily activity
Intensity (how hard?)	Moderate to vigorous^a^	None specified	Moderate to high >40–60 % of maximum	Moderate 40–< 60 % of VO_2reserve_	Moderate^a^	Moderate^a^
Time (how long?)	40 min⋅session^−1^	≥30 min⋅day^−1^	150 min⋅week^−1^	30-60 min continuous or accumulated in bouts ≥10 min each	≥30 min⋅day^−1^	Accumulation of 30–60 min⋅day^−1^
Type (what kind?) *Primary*	Aerobic	Aerobic	Aerobic	Aerobic	Aerobic	Dynamic exercise (Aerobic)
Evidence rating	“High”^b^ Grade B^b^, Class IIa level of evidence A^c^	NA	Class I level of evidence A ^c^	Evidence category A^d,e^ Evidence category B^d,e^	Class I level of evidence A–B^f^	Grade D^g^
*Adjuvant*	NA	NA	Dynamic RT	Dynamic RT 2–3 days⋅week^−1^, moderate 60–80 % of 1-RM, 8–12 repetitions	Dynamic RT 2–3 days⋅week^−1^	Dynamic, Isometric, or Handgrip RT
Evidence rating	NA	NA	Class IIa level of evidence B^c^	Evidence category B^d,h^	NA	Grade D^g^

*Abbr. AHA/ACC* American heart association/American college of cardiology, *FITT* Frequency, Intensity, Time, and Type*, NA* not applicable, *RT* resistance training. VO_2reserve_ oxygen uptake reserve

^a^Moderate intensity, 40–<60 % VO_2reserve_ or an intensity that causes noticeable increases in heart rate and breathing; vigorous or high intensity, ≥60 % VO_2reserve_ or an intensity that causes substantial increases in heart rate and breathing

^b^ *Evidence statement*: “Aerobic exercise lowers blood pressure (BP)” was rated *High* ^d^; *Evidence recommendation* for the FIT to lower BP was rated *grade B* (adapted from [97]) or *Moderate*, corresponding to *Class IIa level of evidence A* ^c^

^c^Guideline criteria from the American Heart Association [19] was used to classify the strength of evidence

^d^Criteria from the National Heart, Lung, and Blood Institute [98] was used to rate the level of evidence

^e^The strength of evidence for aerobic exercise was rated: *category B* ^d^ for its immediate effects (i.e., postexercise hypotension [PEH]); *category A* ^d^ for its long-term (i.e., chronic effects); the FIT to lower BP was rated *category B* ^d^

^f^Criteria from the European Society of Cardiology [99]

^g^Evidence grading was assigned based on the underlying level of evidence [100], where *grade A* is the strongest evidence (i.e., based on high-quality studies) and *grade D* is the weakest evidence (i.e., based on low-power imprecise studies or expert opinion alone); “higher intensity exercise is not more effective” was assigned *grade D*.

^h^The strength of evidence for dynamic RT’s immediate effects (i.e., PEH) was rated *category C* ^c^. Table 1 is adapted from reference [5••]

Despite the general consensus that exercise, particularly aerobic exercise, lowers resting BP, our systematic reviews of 33 meta-analyses on the BP response to exercise [4••] and the existing professional exercise recommendations for hypertension [5••] revealed differences in the recommended components of the Frequency, Intensity, Time, and Type or FITT principle of exercise prescription (Ex R_x_) as well as the reported magnitude of the BP reductions that result from them. Therefore, the purposes of this review are to (1) overview the existing professional exercise recommendations for hypertension in terms of the FITT principle of Ex R_x_; (2) discuss new and emerging research related to the Ex R_x_ for hypertension; and (3) present an updated FITT Ex R_x_ for adults with hypertension from our previous review [22] that integrates the existing recommendations with new and emerging research.

## Systematic Review Methods

**Fig. 1:**
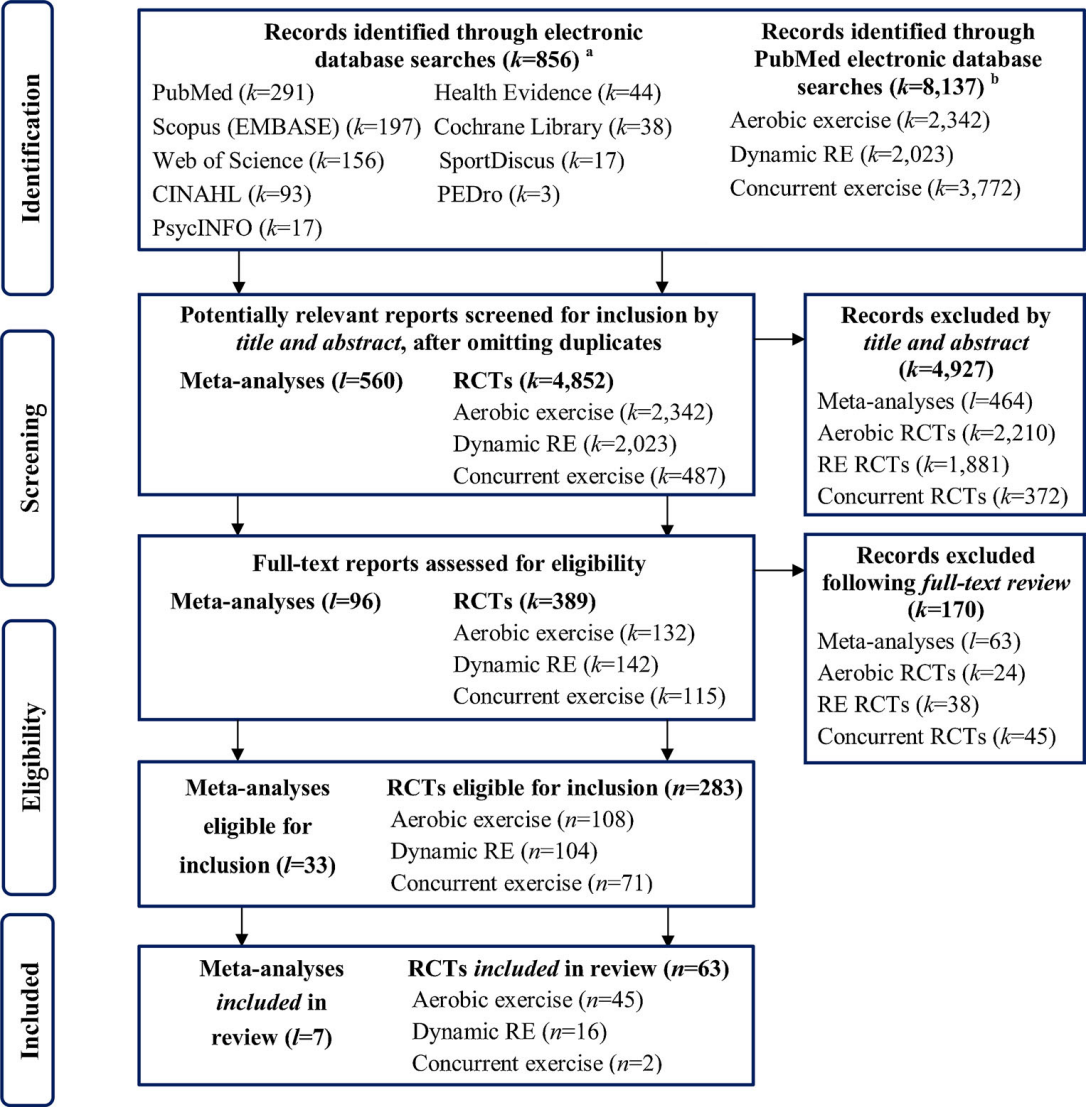
Flow diagram detailing the systematic search for potentially relevant reports (*k*) and the selection process of included meta-analyses (*l*) and exercise trials (*n*). *CINAHL* cumulative index to nursing and allied health literature. *EMBASE*–Excerpta Medica dataBASE. *PEDro* physiotherapy evidence database. *RCTs*—Randomized controlled trials. RE—Resistance exercise. **a** Indicates the databases that were searched in our previous systematic review to locate relevant meta-analyses; the complete search strategy is available from reference [4••]. **b** Indicates the databases that were searched to locate potentially relevant exercise studies published since the ACSM position stand [6]; PubMed also includes the electronic database MEDLINE. Adapted from references [4••, 5••, 43••]

In this review, we have combined and updated the comprehensive search strategies used in our recently published systematic reviews [4••, 5••] to include the potentially relevant literature on the BP response to the acute and chronic aerobic, dynamic resistance, and concurrent exercise since the publication of the ACSM position on exercise and hypertension [6]. The full search details for our systematic reviews have been published elsewhere [4••]. For our updated literature search, studies involving human adults (≥19 years) that were published in English between January 1, 2004 and July 1, 2015, and had a control/comparison group were identified using the electronic database PubMed (including Medline). After omitting duplicates, our combined search yielded 5,412 potential reports, of which 560 were meta-analyses. Overall, 33 meta-analyses and 283 exercise trials were eligible for inclusion. Of those, the authors selected the most relevant meta-analyses (*l* = 7) and exercise studies (*n* = 63) for this review. Figure 1 details the search and selection process of the included meta-analyses and exercise trials.

## The Existing Professional Exercise Recommendations for Hypertension

Prior to overviewing the professional exercise recommendations for hypertension, it is important to define what an Ex R_x_ is as this definition will organize the discussion that follows. An Ex R_x_ is the process whereby the recommended exercise regimen is designed in a systematic and individualized manner in terms of the Frequency (How Often?), Intensity (How Hard?), Time (How Long?), and Type (What Kind?), or the FITT principle of Ex R_x_ [23••]. As previously stated, exercise is recommended as a key lifestyle therapy among adults with hypertension by all professional committees and organizations listed in Table 1. We now overview the existing professional exercise recommendations for hypertension in terms of the FITT principle of Ex R_x_ while commenting on new and emerging research.

### Frequency

As Table 1 shows, all professional committees/organizations recommend exercising on most, if not all, days of the week with the exception of the Lifestyle Work Group [18] that recommended exercising 3–4 days per week for at least 12 weeks among adults with hypertension. Our group and others have shown that the reason exercise should be recommended on most, preferably all days, of the week is because BP is lower on the days people exercise compared to the days they do not exercise. This physiological response is termed *postexercise hypotension* (PEH) [24, 25]. PEH is the immediate reduction in BP of 5–7 mmHg among people with hypertension that occurs after a single, isolated session of aerobic exercise of varying durations (10 to 50 min) and intensities (40 % up to 100 % of maximum oxygen consumption [VO_2max_]), and these BP reductions are sustained for up to 24 h after the exercise bout [6, 26–42, 43••].

The merits of PEH as antihypertensive lifestyle are further supported by two recent studies by Liu et al. [44] and Hecksteden et al. [45] who found that the BP response to acute exercise was strongly correlated with the more long-term BP response to exercise training. These findings support the long held notion that PEH may account for a significant amount of the magnitude of the BP reduction attributed to exercise training [6, 26, 46]. They also suggest that PEH could be used as a health screening tool to identify individuals with hypertension who respond to aerobic exercise as antihypertensive therapy. For individuals determined not to be responsive, alternative forms of treatment can then be more rapidly applied for the treatment and control of their high BP [47]. In fact, Luttrel and Halliwill’s [48] conceptual model of recovery from exercise labeled PEH as a “window of opportunity” that can be exploited as a health screening tool to increase the effectiveness of exercise as antihypertensive lifestyle therapy.

Despite the clinical utility of PEH as antihypertensive therapy, only the ACSM [6] has addressed the merits of PEH by providing graded evidence for the antihypertensive effects of this seemingly important phenomenon. In addition to PEH, another reason for the recommendation of exercising on most, if not all, days of the week is that adults with hypertension are often overweight or obese, and a high frequency (days per week) or volume (metabolic energy equivalents [MET] × minutes per week) of exercise is needed to achieve the caloric expenditure required for initial weight loss and successful maintenance of that weight loss [23••].

### Intensity

The JNC 8 [17] and Lifestyle Work Group [18], AHA [19], ACSM [6], ESH/ESC [20], and CHEP [21] all recommend adults with hypertension engage in moderate intensity aerobic exercise (40 % to <60 % VO_2max_ or heart rate [HR] reserve), whereas the intensity of exercise was not specified by JNC 7 [16]. Of note, the Lifestyle Work Group [18] and AHA [19] also endorse vigorous intensity (≥60 % VO_2max_ or HR reserve) aerobic exercise for people with hypertension. This endorsement of vigorous intensity aerobic exercise incorporates new and emerging evidence from our laboratory, and others, showing that the magnitude of the BP reductions that result from acute and chronic aerobic exercise occur as a direct function of intensity such that the more rigorous the intensity, the greater the resultant BP reductions [8, 23••, 42, 43••, 49–58].

**Fig. 2:**
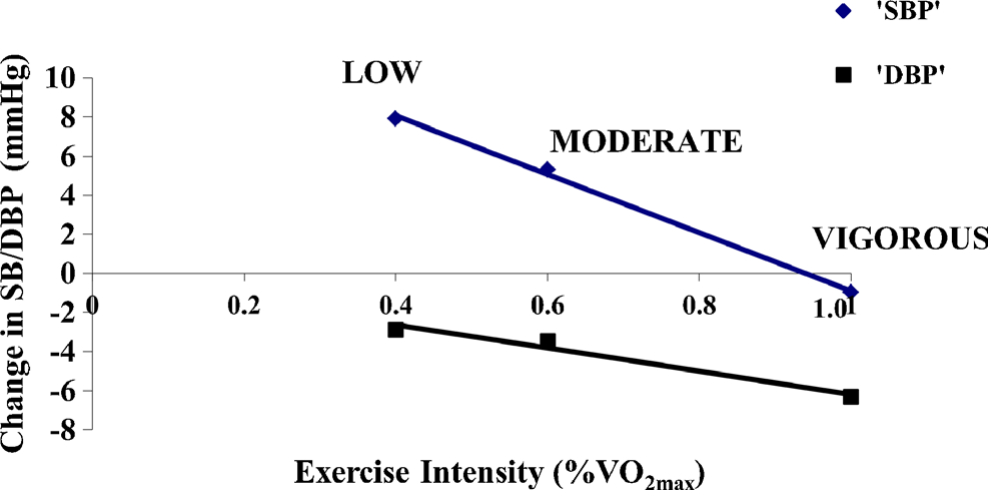
Linear regression of the average blood pressure change from baseline following *low*, *moderate*, and *vigorous* intensity exercise. SBP systolic blood pressure. DBP diastolic blood pressure. VO_2max_ maximum oxygen consumption. *Black diamond suit* indicates SBP, y = -14.9*x* + 14.0, *R* ^*2*^ = 0.998. *Black square* indicates DBP, y = -5.9*x*–0.3, *R* ^*2*^ = 0.969 (*ps* < 0.01). Adapted from reference [42]

Eicher and colleagues [42] examined the antihypertensive effects of three bouts of acute aerobic exercise performed at light (40 % VO_2max_), moderate (60 % VO_2max_), and vigorous (a graded maximal exercise stress test to exhaustion or 100 % VO_2max_) intensity aerobic exercise among 45 middle aged, overweight men with pre- to stage 1 hypertension who were monitored in the laboratory and under ambulatory conditions. Eicher et al. [42] found that for each 10 % increase in relative VO_2max_, SBP decreased 1.5 mmHg (y = -14.9*x* + 14.0, *R*^2^ = 0.998) and DBP 0.6 mmHg (y = -5.9*x*–0.3, *R*^2^ = 0.969) over the course of the day time hours (Fig. 2). These findings suggest more vigorous levels of acute physical exertion lower BP to greater levels than lower levels of physical exertion among adults with hypertension who are willing and able to tolerate more intense levels of exercise.

High intensity interval training (HIIT) is defined as alternating periods of brief, very high intensity aerobic exercise (>90 % VO_2max_) separated by recovery periods of lower intensity exercise or rest [51]. Consistent with Eicher et al.’s findings [42], several investigators have found HIIT to be superior to continuous, moderate intensity aerobic exercise training for eliciting improvements in CVD risk factors when training programs were matched for exercise volume among a variety of populations, including adults with coronary artery disease, congestive heart failure, the metabolic syndrome, and overweight and obesity [49, 50, 56, 57]. Furthermore, the magnitude of the BP reductions following HIIT was greater among samples with higher resting BP; ∼8 mmHg for hypertension [49] and prehypertension [50] versus ∼3 mmHg for normal BP [56]. These findings are consistent with the law of initial values that BP will be lowered to the greatest levels among those with higher resting BP [26]. Collectively, these new and emerging findings [42, 43••, 49, 50, 56, 57] indicate that exercise intensity is an important determinant of the BP response to exercise such that increasing levels of physical exertion appear to lower BP in a dose–response pattern.

Holloway and colleagues recently examined the skeletal muscle [58] and cardiac [59] adaptations to 4 weeks of HIIT compared to traditional moderate intensity aerobic exercise training among Dahl salt-sensitive rats, an animal model of hypertension. They found HIIT had a negative impact on cardiac function and the overall oxidative capacity of skeletal muscle among the rats with hypertension, whereas moderate intensity aerobic exercise resulted in favorable cardiac and skeletal muscle adaptations. These provocative findings, in addition to the fact that adults with hypertension are predisposed to a transient increase in cardiovascular risk upon sudden vigorous exertion [60–62], highlight the need for further investigation to determine the benefit-to-risk ratio of exercising at vigorous intensity among adults with hypertension before the current recommendations in Table 1 can be expanded to include vigorous intensity exercise.

### Time

All professional organizations and committees recommend exercising at least 30 min per day among people with hypertension. Consistent with the general consensus of most, preferably all, days of the week for the frequency recommendation, there is also a high level agreement among the professional organizations and committees in Table 1 that the duration of exercise should achieve a total of 150 or more minutes per week; an amount that is consistent with the recommendations for the general population [23••, 63, 64].

There is emerging evidence that acute aerobic exercise performed continuously in a single bout or accumulated in shorter bouts throughout the day can lower BP to similar levels and durations among adults with hypertension [43••, 55, 65–70]. Guidry et al. [71] compared the effects of a short (15 min) and long (30 min) acute aerobic exercise bout performed at light (40 % VO_2max_) or moderate (60 % VO_2max_) intensity on PEH among 45 white, middle-aged overweight men with pre- to stage 1 hypertension. They found an acute bout of aerobic exercise performed for as short as 15 min at light to moderate intensity resulted in PEH for the remainder of the day [71]. In addition, Ciolac and co-investigators [72] randomized 52 men and women on antihypertensive medication to either 40 min of acute aerobic exercise performed continuously at 60 % HR reserve or an interval aerobic exercise session that alternated between 2 min at 50 % HR reserve and 1 min at 80 % HR reserve to total 40 min. The continuous exercise group lowered ambulatory SBP and DBP 4–8 mmHg, while the interval exercise group lowered ambulatory SBP only 5–6 mmHg over 24 h. Finally, Bhammar and colleagues [73] compared the effects of fractionized aerobic exercise (three 10-min bouts) interspersed throughout the day (morning, midday, and afternoon) and continuous aerobic exercise (one 30-min bout) performed at 60–65 % VO_2max_ on ambulatory BP among 11 young subjects with prehypertension. They found fractionized exercise was as at least as effective as continuous exercise in eliciting PEH until the following morning. Miyashita and colleagues [66] found that even shorter bouts of aerobic exercise (10 3-min bouts) interspersed throughout the day were as effective as a 30-min bout of continuous aerobic exercise in eliciting PEH.

Collectively, these findings [66, 71–73] and others [67–70, 74] support that PEH is a low threshold phenomenon in terms of the duration of the exercise bout needed to produce the effect; and when these short bouts of exercise are interspersed throughout the day, PEH is a viable therapeutic lifestyle option for BP control among individuals with high BP. Not having the time to exercise is often a major deterrent to starting and maintaining a regular exercise program. For this reason, performing accumulated, shorter exercise bouts throughout the day (i.e., 3 to 10 min to total 30 min or more) would appear to be an attractive therapeutic option among adults with hypertension [43••, 65–74]. Nonetheless, future research is needed to determine if interspersing shorter bouts of aerobic exercise throughout the day may be used as a behavioral strategy to increase exercise adherence in this population.

### Type

There is broad consensus supported by a strong rating of evidence that aerobic exercise should be prescribed as the primary type of exercise for the prevention, treatment, and control of hypertension. This recommendation is made by all professional organizations and committees in Table 1 because aerobic exercise training has been consistently shown to lower BP 5–7 mmHg among those with hypertension, levels that are twice that resulting from dynamic resistance training [6, 16–21]. The AHA [19], ACSM [6], ESH/ESC [20], and CHEP [21] recommend that adults with hypertension engage in dynamic resistance training as a supplement to aerobic exercise training, while the JNC 7 [16], JNC 8 [17], and Lifestyle Work Group [18] did not make any specific recommendations regarding dynamic resistance training.

As Table 1 shows, the level of evidence upon which the dynamic resistance training recommendations are made is weak, which may contribute to the lack of consensus among professional organizations and committees regarding the effectiveness of dynamic resistance training as antihypertensive therapy. One possible reason for this weak rating of evidence may be partially attributed to the dearth of primary level studies investigating dynamic resistance training as antihypertensive lifestyle therapy among adults with hypertension. This short-coming likely underestimates the effectiveness of dynamic resistance exercise training as antihypertensive lifestyle therapy due to the law of initial values, which predicts that the largest BP reductions would occur in adults with hypertension [5••, 6–8, 13, 26].

Indeed, several primary level studies have shown that the BP reductions following dynamic resistance training may be comparable in magnitude to those that result from aerobic exercise training among adults with high BP [43••, 75–84]. Mota and colleagues [84] found 16 weeks of moderate intensity dynamic resistance training reduced SBP/DBP about 14/4 mmHg among 32 older women with controlled hypertension. Moraes et al. [85] found 12 weeks of moderate intensity dynamic resistance training reduced SBP/DBP approximately 16/12 mmHg among 15 middle-aged men with hypertension. In addition, several controlled trials [43••, 50, 76–78, 81, 86–88] directly comparing the effectiveness of aerobic exercise training versus dynamic resistance training as antihypertensive therapy found that SBP/DBP were reduced to similar levels among adults with untreated [76, 77] and controlled hypertension [78, 81], with no statistical difference between modalities. BP reductions of this magnitude following dynamic resistance training have also been reported among young [50, 86, 89] and middle-aged [87, 88, 90] adults with prehypertension. These findings suggest that moderate intensity dynamic resistance training may be viable as stand-alone antihypertensive lifestyle therapy among adults with hypertension. Nonetheless, more RCTs are needed to more definitively determine whether the existing professional exercise recommendations for hypertension should be expanded to include dynamic resistance training as stand-alone lifestyle therapy, and more precisely define for what patient populations and FIT features of dynamic resistance training programs would elicit the greatest BP benefits.

Last, it is not well understood how the combined effects of aerobic exercise and dynamic resistance training, termed *concurrent exercise training*, influence resting BP among adults with hypertension. Concurrent exercise training is defined as aerobic and dynamic resistance training performed in close proximity to each other (i.e., in a single session or on separate days) [43••, 91–93]. In light of evidence suggesting that dynamic resistance training may be as effective as aerobic exercise training as stand-alone antihypertensive lifestyle therapy among those with hypertension, the antihypertensive effects of concurrent exercise training are worthy of mention [5••, 43••].

Hayashino et al. [94] performed a meta-analysis of 42 trials, of which 14 were concurrent exercise training trials. Overall, the sample included middle-aged adults with type 2 diabetes mellitus and about 36 % had hypertension. The authors [94] reported SBP/DBP reductions following aerobic exercise training of 1.7/2.3 mmHg, dynamic resistance training of 2.8/2.3 mmHg, and concurrent exercise training of 3.2/1.9 mmHg, BP reductions that were not different among the three modalities of exercise. Furthermore, Cornelissen and Smart [8] found in a sample of 93 trials, of which 14 included concurrent exercise training trials, BP was reduced 3.5/2.5 mmHg following aerobic exercise training, 1.8/3.2 mmHg following dynamic resistance training, and 2.2 mmHg (SBP only) following concurrent exercise training, and once again, the BP reductions were not different among the three modality groups. Clearly, further investigation is needed to explore the promising merits of concurrent exercise training as antihypertensive lifestyle therapy.

## An Exercise Prescription for Hypertension Update

The FITT Ex R_x_ recommendations that follow are based upon the existing exercise recommendations for hypertension displayed in Table 1, while integrating the new and emerging research we have discussed in this review.

### Frequency

Aerobic exercise on most, preferably all days of the week and dynamic resistance exercise on 2 to 3 days in that same week.

### Intensity

Moderate intensity aerobic exercise (i.e., 40 to <60 % VO_2max_ or HR reserve; 11–13 rating of perceived exertion [RPE] on the 6–20 Borg Scale [95, 96]) and moderate intensity dynamic resistance exercise (60 % to 80 % one repetition maximum [1-RM]).

Due to emerging evidence that the BP reductions resulting from exercise are dose-dependent upon the intensity of exercise [42, 43••, 49–52, 56, 57, 72], the intensity recommendation may be expanded in the future to include vigorous intensity pending the results of future research that better establishes the benefits and risks of more rigorous levels of exercise among those with hypertension.

### Time

Aerobic exercise should be performed for 30 to 60 min per day that is continuous or accumulated. If accumulated, bouts should be at least 10 min in duration to total 30 to 60 min of exercise per day. Dynamic resistance exercise should consist of two to three sets of 10 to 12 repetitions for 8 to 10 exercises that target the major muscle groups of the upper and lower body. The duration of exercise should total 150 min or more per week.

### Type

Examples of aerobic activities may include walking, jogging, cycling, and swimming. Dynamic resistance training equipment may include machine weights, free weights, and resistance bands, as well as functional body weight exercises.

Due to evidence supporting the merits of both dynamic resistance [75–84] and concurrent exercise training [8, 43••, 94], it seems prudent that adults with hypertension should perform combinations of aerobic and dynamic resistance exercise during a given week. However, due to the weak and limited nature of this literature (Table 1) [4••, 5••], further research is needed to explore the merits of dynamic resistance and concurrent exercise training as antihypertensive therapy.

### Progression

The FITT principle of Ex R_x_ relating to progression for healthy adults generally applies to those with hypertension [63]. Progression should be gradual, avoiding large increases in any of the FITT components of the Ex R_x_, especially intensity [23••]. Health care and exercise professionals should also consider the level of BP control, recent changes in antihypertensive drug therapy, medication-related adverse and exercise effects, and the presence of target organ disease and/or other comorbidities with adjustments made accordingly [5••, 6, 23••].

## Conclusion

Hypertension is arguably one of the most important CVD risk factors due to its high prevalence and medical costs [1]. Indeed, nearly 70 % of Americans have pre- to established hypertension. Aerobic exercise is universally recommended as initial lifestyle therapy for individuals with hypertension because it lowers BP 5–7 mmHg among adults with hypertension. Nonetheless, the components of the FITT principle of Ex R_x_ differ among the existent recommendations [5••]. Considering both the exercise recommendations for hypertension in Table 1 and new and emerging literature, we have formulated an updated FITT Ex R_x_ from our previous review [22] as follows: a combination of 30 min or more per day of moderate intensity aerobic exercise on most, preferably all, days of the week and dynamic resistance exercise 2 to 3 days per week to total 150 min or more of exercise per week. The notable difference in this updated FITT Ex R_x_ from our previous review is a greater emphasis on inclusion of dynamic resistance exercise in combination with aerobic exercise. Further investigation is needed to more precisely establish the FIT combinations of aerobic and resistance exercise that elicit the greatest BP benefit among adults with hypertension.
